# Creatine Supplementation, Physical Exercise and Oxidative Stress Markers: A Review of the Mechanisms and Effectiveness

**DOI:** 10.3390/nu13030869

**Published:** 2021-03-06

**Authors:** Hamid Arazi, Ehsan Eghbali, Katsuhiko Suzuki

**Affiliations:** 1 Department of Exercise Physiology, Faculty of Sport Sciences, University of Guilan, Rasht 4199843653, Iran; ehsan.eghbali1990@gmail.com; 2 Faculty of Sport Sciences, Waseda University, Tokorozawa 359-1192, Japan

**Keywords:** reactive oxygen species, creatine supplementation, exercise, antioxidants

## Abstract

Oxidative stress is the result of an imbalance between the generation of reactive oxygen species (ROS) and their elimination by antioxidant mechanisms. ROS degrade biogenic substances such as deoxyribonucleic acid, lipids, and proteins, which in turn may lead to oxidative tissue damage. One of the physiological conditions currently associated with enhanced oxidative stress is exercise. Although a period of intense training may cause oxidative damage to muscle fibers, regular exercise helps increase the cells’ ability to reduce the ROS over-accumulation. Regular moderate-intensity exercise has been shown to increase antioxidant defense. Endogenous antioxidants cannot completely prevent oxidative damage under the physiological and pathological conditions (intense exercise and exercise at altitude). These conditions may disturb the endogenous antioxidant balance and increase oxidative stress. In this case, the use of antioxidant supplements such as creatine can have positive effects on the antioxidant system. Creatine is made up of two essential amino acids, arginine and methionine, and one non-essential amino acid, glycine. The exact action mechanism of creatine as an antioxidant is not known. However, it has been shown to increase the activity of antioxidant enzymes and the capability to eliminate ROS and reactive nitrogen species (RNS). It seems that the antioxidant effects of creatine may be due to various mechanisms such as its indirect (i.e., increased or normalized cell energy status) and direct (i.e., maintaining mitochondrial integrity) mechanisms. Creatine supplement consumption may have a synergistic effect with training, but the intensity and duration of training can play an important role in the antioxidant activity. In this study, the researchers attempted to review the literature on the effects of creatine supplementation and physical exercise on oxidative stress.

## 1. Introduction

Many athletes have utilized ergogenic aids to maintain fitness, improve recovery, and physiological adaptations in long-term exercise programs. Therefore, the effects of ergogenic aids have always attracted a lot of attention, and many researchers have tried to combine exercise programs and ergogenic aids to enhance the benefits of exercise [1,2].

One of the favorite ergogenic supplements among athletes (at all levels) is creatine. Studies have shown that creatine supplementation combined with resistance training had a higher effectiveness in training and increased muscle strength and lean mass [1,2]. As a popular creatine supplement in the sports and fitness industry, it is believed that creatine supplementation helps maintain high-energy phosphate stores during exercise. Moreover, specific mechanisms of creatine supplementation have been identified in improving athletic performance [3,4]; there are ambiguities about its effects on oxidative stress and its mechanism of action. The antioxidant effects of creatine may be due to various functional mechanisms, such as indirect mechanisms involved in the cell membrane stabilization and improvement of cellular energy capacity [5] and its direct antioxidant properties [6]. Oxidative stress reduces strength and performance [7]; mechanically, reactive oxygen species (ROS) can speed up skeletal muscle fatigue by reducing calcium sensitivity [8] and can decrease maximal calcium-activated force [9]. ROS are free radical molecules that can oxidatively alter cellular compounds such as lipids, proteins, and DNA, and damage cells [10]. They are also associated with several diseases such as cancer, cardiovascular disease, Parkinson’s, Alzheimer’s, etc. [11]. Increased ROS production due to certain diseases or exercise can exceed the capacity of the antioxidant system, which can lead to oxidative stress and dysfunction. However, its predominant impact on the human health and function is still controversial [12].

One of the common physiological conditions associated with the enhancement of oxidative stress is exercise [13,14]. Exercise can have positive and negative effects on oxidative stress [15]. High-intensity exercise can lead to a temporary imbalance between the active oxygen/nitrogen species production and removal, which can lead to oxidative stress. Although exercise-induced ROS is required for the production of natural force in the muscles, high levels of ROS appear to cause contractile dysfunction [16]. Exercise-induced ROS production is important for exercise-induced mitochondrial biogenesis [17], because ROS are used as signaling molecules to activate redox-sensitive signaling pathways [16]. Evidence suggests that exercise intensity and duration are associated with oxidative stress in humans, and has been confirmed by several studies [18,19]. Intense exercise or exercise in untrained people is associated with a greater increase in oxidative stress compared to moderate and regular aerobic exercise [20]. In addition, long-term regular training may improve some antioxidant defense mechanisms, and thus may limit mitochondrial oxidative damage [21,22].

Using antioxidant supplements along with physical activity can reduce the harmful effects of oxidative stress caused by exercise, increase the antioxidant defense system associated with exercise and increase the positive effects of physical activity. Creatine is one of the most popular supplements for athletes; it can act as a cellular energy buffer, increase creatine phosphate (CrP) and adenosine triphosphate (ATP) regeneration [23]; additionally, creatine compounds can have different effects. It seems that creatine has significant antioxidant effects. In general, the purpose of this study was to investigate the available information on the effects of endurance, resistance, and combination exercise along with the creatine supplementation on oxidative stress and their mechanism of action. Therefore, the present study tried to summarize the available information and research on the effects of creatine supplement consumption and physical exercise on oxidative stress and how it works, together with the mechanisms of action.

## 2. Materials and Methods

**Figure 1:**
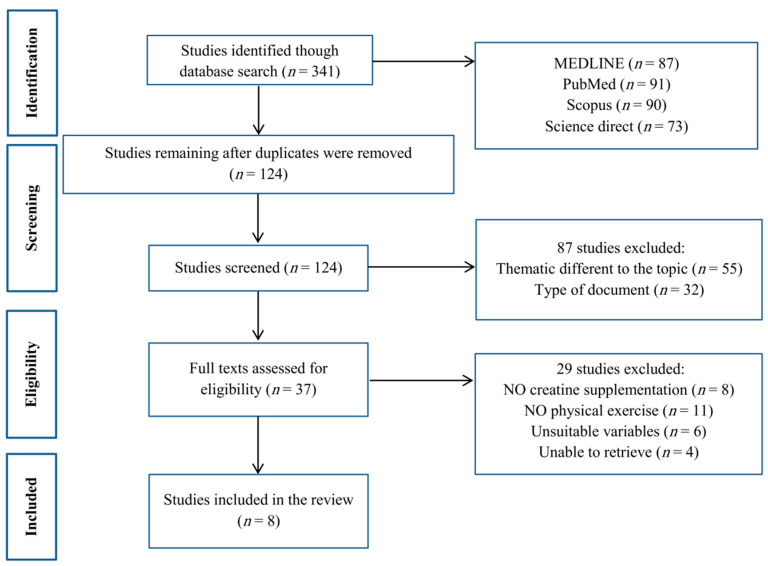
Flowchart of the study selection.

According to the purpose of the research, a search was performed in MEDLINE, PubMed, Scopus, the Directory of Open Access Journals and Science Direct databases; the keywords used included physical exercise, creatine supplementation, and oxidative stress. Our focus was on English articles published from 2008 to 2020. As shown in Figure 1, in the first stage, 341 articles were reviewed, and after removing duplicate articles and articles unrelated to the research objectives in several stages, 8 articles were selected for full study of their findings. After selecting articles and reviewing their findings on physical activity and creatine supplementation, information was collected focusing on the research objectives (Figure 1).

## 3. Oxidative Stress

Oxidative stress is an enclosed physiological pathway regulated by the antioxidant mechanisms. Improper regulation of oxidative stress is correlated with several recurrent pathological or physiological conditions [14]. Oxidative stress can be defined as an imbalance between the generation of harmful free radicals and their removal by the antioxidant defense system. Highly reactive unstable free radicals are composed of many compounds. However, the most common are ROS (superoxide, hydroxile, alcoxile, peroxile, hydrogen peroxide) and reactive nitrogen species (RNS) (nitric oxide, nitrogen dioxide, peroxinitrile); collectively called reactive oxygen and nitrogen species (RONS) [24]. Free radicals are very reactive atoms or molecules that have one or more unpaired electrons in their outer shell and can be formed by the interaction of oxygen with specific molecules [25]. These radicals are produced by the loss or acceptance of an electron in cells, therefore behaving as the oxidants or reductants [26]. The endogenous sources of RONS consist of: nicotinamide adenine dinucleotide phosphate (NADPH) oxidase, myeloperoxidase (MPO), lipoxygenase, and angiotensin II [27]. External sources of RONS are air pollution, tobacco, alcohol, drugs, industrial solvents, etc., which are metabolized to free radicals in the body [28].

NADPH oxidase is a common source of the superoxide anion (O_2_^−^) formed by the reduction of one electron of the molecular oxygen by electrons supplied by the NADPH during cellular respiration. Most O_2_^−^ is catalyzed by superoxide dismutase (SOD) to hydrogen peroxide (H_2_O_2_) [29]. H_2_O_2_ is not a free radical because it has no unpaired electrons, but via the Fenton or Haber-Weiss reaction, it is able to form very reactive hydroxyl radicals (OH^−^). Hydroxyl radicals are highly reactive and react especially with phospholipids in cell membranes and proteins [30].

Too much RONS can cause irreversible damage to the biological molecules, proteins, carbohydrates, lipids, RNA and DNA, leading to the spread of many pathological problems and oxidative tissue damage [31]. Antioxidant systems suitable with enzymatic (e.g., SOD, catalase (CAT) and glutathione peroxidase (GPX)) and non-enzymatic (e.g., uric acid, bilirubin, vitamin E, vitamin C, glutathione (GSH), ascorbic acid, and α-tocopherol) processes, both act to reduce the oxidation potential of RONS through direct and indirect mechanisms. Direct antioxidants activate redox reactions and trap and inactivate RONS in a process that is sacrificed and must be regenerated [32]. On the other hand, indirect antioxidants may or may not be redox-active and apply their antioxidant effects via the up-regulation of cytoprotective proteins [32].

The ROS involve many physiological functions. The intracellular concentration of ROS increases transiently in response to a stimulus such as cytokines, growth factors, or other hormones; this pattern is common in many physiological conditions, where the release of ROS is quickly controlled by the antioxidant regulatory mechanisms. If stable or unbalanced, increased oxidative stress may suppress antioxidant capabilities, and the ROS can cause damage [14]. The ROS release is involved in major cellular signaling pathways and allows the transmission of extracellular stimuli and changes in cell physiology by modulating the transcription of some genes or by post-transcriptional modulation. To date, “redox-responsive” signaling pathways have been implicated in important functions such as nitric oxide (NO) generation, vascular tone regulation and neurotransmission, cell adhesion, immune responses, and hypoxia and apoptosis [11,33].

## 4. Creatine and Oxidative Stress

**Figure 2:**
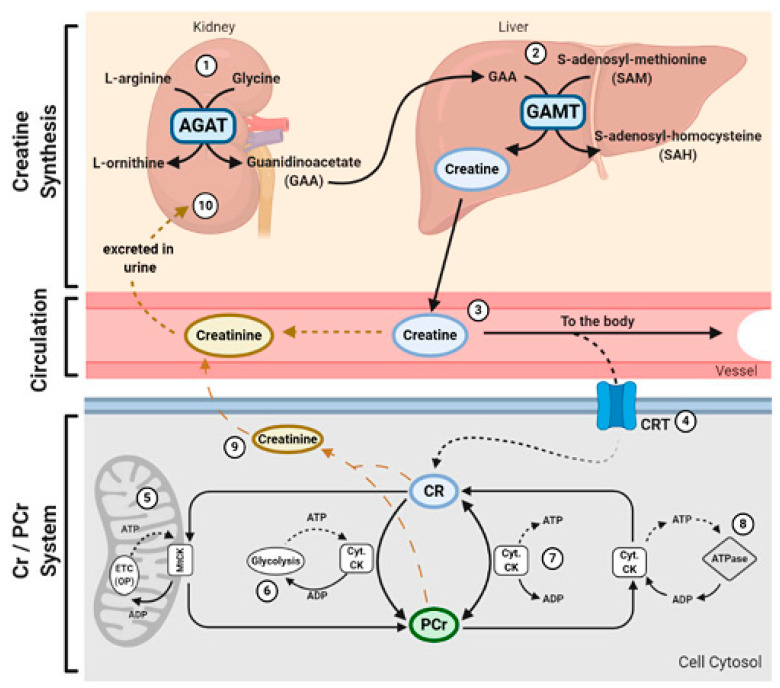
Physiological structure of creatine. Adopted from Clarke et al. [41]. L-arginine: glycine amidinotransferase (AGAT); anidinoacetate N-methyltransferase (GAMT); phosphocreatine (PCr); cytosolic creatine kinase (Cyt. CK); electron transport chain (ETC); adenosine diphosphate (ADP); mitochondrial creatine kinase (mtCK); and adenosine triphosphate (ATP).

Creatine is a metabolite of three amino acids (arginine, glycine, and methionine) that are synthesized by the cooperation of various organs, including the liver, pancreas, and kidneys [34]. Beef is a rich source of arginine, glycine and methionine. In contrast, all plant-based foods contain small amounts of glycine and methionine, and most plant foods (except soy, peanuts, and other nuts) also contain small amounts of arginine [35]. The beginning of creatine synthesis is by arginine; the guanidino group from arginine to glycine is transferred by glycine amidinotransferase, and produces guanidinoacetate and ornithine (Figure 2). It seems that the arginine–glycine aminotransferase is fundamentally expressed in the kidney tubules, pancreas, and a little in the liver and other organs. Thus, guanidineacetate is produced by renal components. The guanidinoacetate released by the kidneys is methylated by guanidinoacetate N-methyltransferase, which is mainly found in the liver, pancreas, and to a very small extent in the kidneys, and produces creatine [36].

Creatine synthesis is primarily regulated as follows: (1) changes in the renal arginine expression: glycine aminotransferase in rats and humans; and (2) the availability of substrates. Dietary creatine intake and circulating growth hormone (GH) levels are major factors influencing new creatine synthesis [36]. Creatine supplements and GHs do not affect the hepatic activity of guanidinoacetate N-methyltransferase in animals. Thus, a creatine supplement helps to store arginine, glycine, and methionine for use through other vital metabolic pathways such as protein synthesis, NO, and glutathione. This is of great nutritional and physiological importance [34,37].

Studies have shown that creatine supplementation can have antioxidant properties. The first evidence of creatine-like antioxidant activity was reported by Matthews et al. [13]. They stated that creatine supplementation could protect rats against nitropropionic acid intoxication (an animal model of Huntington’s disease). Moreover, Hosamani et al. showed a reduction in mitochondrial oxidative damage induced by rotenone and neurotoxicty in Drosophila melanogaster when supplemented with creatine [38]. The exact mechanism of action of creatine antioxidant is not known. However, it has been shown to increase the activity of antioxidant enzymes and the capability to eliminate ROS and RONS [6,13,39]. Furthermore, 90% of the body’s total creatine is stored in the skeletal muscle, and mitochondria are an important source of ROS, which includes H_2_O_2_ and O_2_^−^, and possibly OH^−^ and peroxynitrite in the skeletal muscle [40].

Creatine protects two different and important cellular targets, mitochondrial deoxyribonucleic acid (mtDNA) and RNA against oxidative damage. In addition, creatine has been shown to cause other related effects that help the cell to survive and function under oxidative stress. Creatine possibly maintains mitochondrial integrity via organelle-directed antioxidant activity [42], which promotes adequate mitochondrialogenesis, and provides a significant amount of thiol contents intracellularly, preventing the RNA from oxidative damage in situations where robust messenger ribonucleic acid (mRNA) use is required and thus exerts its antioxidant effects [42]. Mitochondria and mtDNA are important targets for oxidative damage. Indeed, it has been reported that mtDNA mutations work as an etiologic factor in oxidative stress-related disorders [43], including cardiovascular disease, inherited or acquired neurological disorders, and various types of tumors. Mitochondrial antioxidants have been proposed as a valuable tool to protect mitochondria against pathological changes [44]. Studies have shown that creatine significantly protects mtDNA from oxidative damage [42]. Creatine probably prevents damage through direct antioxidant activity. Thus, its supplementation can play a significant role in genome stability, which can normalize mitochondrial mutagenesis and intercept its functional consequences such as reduced oxygen consumption, mitochondrial membrane potential, ATP content, and cell survival [45,46].

Furthermore, RNA molecules interfere with all stages of gene expression and several other biological activities. RNA damage can also affect the balance between protein breakdown and synthesis and the repair and regeneration processes in the skeletal muscle that ultimately determine muscle mass [46]. RNA damage can be related to exposure to xenobiotics [46]. The protective effect of creatine against doxorubicin activity, which causes RNA damage, can be attributed in part to the production of CrP sources that increase ATP regeneration. Creatine’s protective activity against radicals also points to its role as an antioxidant [47]. Creatine also increases the expression of myogenic transcriptional regulators (MRFs) and IGF-1 mRNAs [48,49] and increases CrP sources [50]. In the case of non-muscle tissue, empirical reports suggested that creatine mightplay a significant role in the differentiation and function of the central nervous system (CNS) neurons. For example, creatine can act as an exocytosis transmitter by nerve cells [51] and adjust gamma-aminobutyric acid (GABA) receptors (inhibitory [52] or stimulant [53]). It is worth noting that the GABA receptor activity plays a main role in the neuronal differentiation [46]. A study by Young et al. showed that mitochondrial reductase and cytoplasm (peroxiredoxin-4, a type 2 peroxiredoxin 2 and thioredoxin-dependent peroxide reductase) were increased in the creatine-treated cells [54]. Incremental regulation of these enzymes may also effectively help several protective effects. Studies have shown that creatine helps cells function and survive under oxidative stress, especially in the differentiation of myoblasts [46].

**Figure 3:**
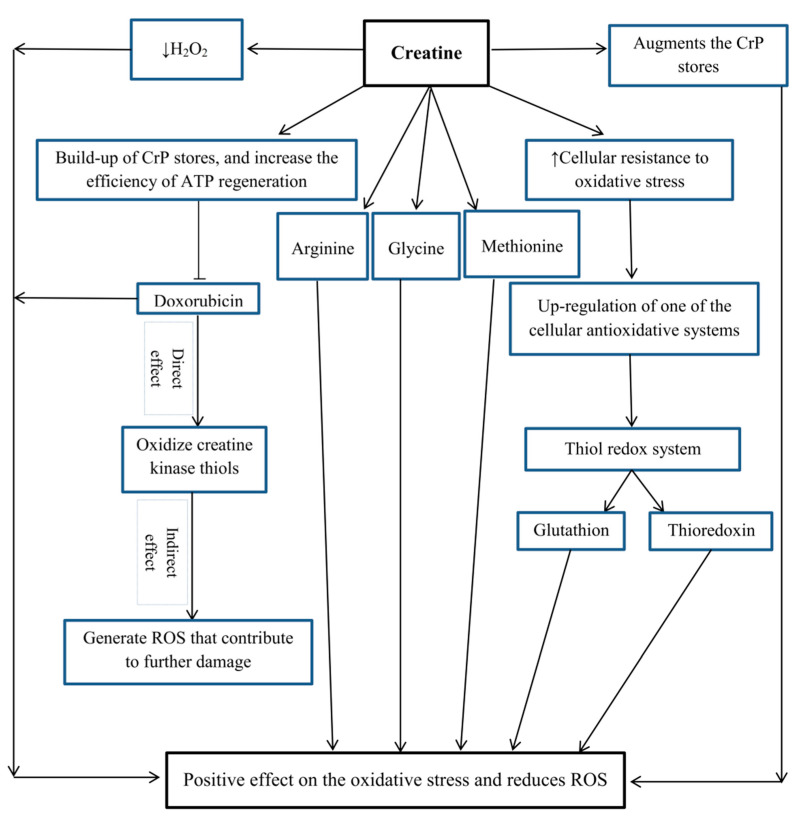
The effect of creatine on oxidative stress. Hydrogen peroxide (H_2_O_2_); creatine phosphate (CrP); adenosine triphosphate (ATP); and reactive oxygen species (ROS).

In addition, the antioxidant properties of creatine may be related to the presence of arginine in its molecule. Arginine is a substrate of the NO synthase family and can enhance NO generation (a free radical that modulates metabolism, contraction, and glucose uptake into the skeletal muscle) [55]. Other amino acids such as glycine and methionine may be particularly sensitive to the oxidation of free radicals due to the presence of sulfhydryl groups (Figure 3) [56].

## 5. The Influence of the Physical Exercise on Oxidative Stress

Physical exercises are usually divided into two major groups: endurance exercise and resistance exercise. Endurance or intense aerobic exercise is commonly known to stimulate ROS and overproduce active nitrogen species due to the increased metabolism, leading to oxidative stress and related injuries [57]. Aerobic exercise is estimated to increase O_2_^−^ 1–3-fold during muscle contraction [58]. However, mitochondria account for only a small fraction of O_2_^−^ production during aerobic exercise [58,59]. In fact, mitochondrial-derived O_2_^−^ formation in the skeletal muscle decreases during the exercise relative to the rest. This is because contractile activity changes the redox state in the muscles to a more oxidative state and reduces the NADH/NAD ratio in the mitochondria. Decrease in the NADH/NAD ratio is related to decreased release of I-dependent O_2_^−^ [58]. During the endurance exercise, ATP is broken down into adenosine diphosphate (ADP) to release energy and support continuous muscle contraction. In some situations, adenosine monophosphate (AMP) is formed, and by a biochemical process involving xanthine oxidase (XO) it can be broken down into hypoxanthine, xanthine, and uric acid. The XO, using molecular oxygen, stimulates the formation of O_2_^−^ and thus exacerbates oxidative stress [60]. In addition, special precautions should be taken for exercise in people with conditions such as asthma; asthma can cause significant ROS and oxidative stress, therefore it can jeopardize the benefits of exercise [61].

Although a period of intense aerobic training may cause oxidative damage to muscle fibers, regular aerobic exercise helps increase the cells’ ability to reduce ROS over-accumulation [62]. Regular moderate-intensity exercise has been shown to increase the activity of endogenous antioxidant enzymes such as SOD, GPX, and CAT [63]. The body’s protection facing chronic low-to-moderate ROS exposure occurs via exercise through elementary conditioning relevant to the redox consisting of repair systems acting as the oxidative damage [62,64]. This adaptation through moderate-intensity exercise also includes an increase in the myocellular antioxidant capacity, which helps reduce the ROS levels [65]. In addition, increasing the ROS formation in the active skeletal muscle by modulating muscle contraction plays an essential role in the adaptation to exercise [62,63]. For example, endurance running is considered important for survival in human development because it can stimulate exercise-related contractile responses through metabolic and redox challenges [62,66]. However, current lifestyles caused reduced physical activity and inhibits human adaptation capacity in redox metabolism and homeostasis [62]. Basic evidence has shown that at least 30 min of exercise (moderate intensity) each day is essential to maintain good health and decrease the potential risks of disease [65].

Accordingly, Zarrindast et al. stated that moderate-intensity aerobic training for eight weeks on the land and water reduces oxidative stress and improves antioxidant status [67]. Moreover, Done et al. concluded that regular aerobic exercise increases resistance to oxidative stress [68]. Estébanez et al. showed that aerobic exercise does not cause significant changes in the oxidative stress biomarkers among the elderly [69]. In addition, Leelarungrayub et al. reported that moderate-intensity aerobic dance for six weeks could reduce malondialdehyde (MDA) and increase total antioxidant capacity (TAC) among inactive women [70]. In general, moderate to intermittent ROS production during a short period of aerobic training program can activate signaling pathways that lead to cellular adaptation and protection against subsequent stresses. In contrast, moderate levels of ROS production over a long period of time (e.g., several hours) or high levels generated during high-intensity short-term training can lead to tissue and structural damage [69].

Despite the need for less oxygen during resistance activities compared to aerobic exercise, the generation of free radicals during the resistance exercise is significant and results from the XO pathway, respiratory burst of neutrophils, catecholamine autoxidation, local muscle ischemia and conversion of weak superoxide to powerful hydroxyl radical with lactate which causes oxidative stress [71,72]. In the case of skeletal muscle, it has been shown that increased ROS formation may impair cellular redox status and lead to the attack of macrophages and other phagocytes, culminating in tissue damage and impaired muscle function [19,73]. Evidence suggests that oxidative damage to biomolecules in cells during acute myeloid leukemia leads to a continuous enhancement in ROS levels and a reduction in the antioxidant cellular defense [74]. Skeletal muscle and myogenic cells are equipped with antioxidants. The antioxidant system inactivates excess ROS/RNS, causing myogenic regeneration and affecting inflammatory reactions, thus stimulating angiogenesis and reducing fibrosis [75]. The oxidative stress-responsive muscle cells include: nuclear factor kappa B (NF-κB), activator protein 1 (AP-1), Nrf2, and peroxisome proliferator-activated receptor gamma coactivator-1 alpha (PGC-1α) [76]. The main role of ROS in the skeletal muscle has been confirmed, both in physiological processes and in fatigue and muscle wasting, aging, and excessive exercise [73]. Skeletal muscle is the biggest tissue in the human body; this system, like other systems, requires the severe regulation of redox homeostasis, such as energy requirements, calcium signaling, and glucose uptake [76]. Skeletal muscle consumes large amounts of molecular oxygen and can produce large amounts of ROS [77].

Resistance training increases the activity of antioxidant enzymes if performed regularly for a long time [78,79]. In this regard, da Silva et al. stated that six months of resistance training can improve people’s response to oxidative stress and this mechanism maybe help better performance and health. Their results showed an increase in the CAT activity and no change in the SOD activity [80]. Furthermore, Vezzoli et al. concluded that 12 weeks of moderate-intensity resistance training can minimize the generation of ROS and oxidative stress. They stated that moderate-intensity resistance training can overcome anabolic resistance and maximize protein synthesis in older adults [81]. In the case of acute resistance exercise, Motameni et al. showed that three types of resistance exercise (hypertrophy, strength, and power) did not worsen oxidative stress in women who practiced resistance exercise. They did not observe a significant change in H_2_O_2_ and MDA levels due to the resistance training [82]. In contrast, they reported that plasma MDA levels had increased after three sets of resistance exercises in untrained men [83]. Based on the evidence, variations of training intensity and volume, or both (high volume-low intensity or low volume-high intensity training) likely have a positive influence on the elevation of GSH concentration [84].

No research has been conducted as of yet on the effects of order of exercise (first strength or endurance exercises) with concurrent exercises on oxidative stress, and it is not clear how they affect it; the need for research in this field is felt. However, in research on the benefits of strength–endurance or endurance–strength training, the results showed that endurance–strength training increases aerobic capacity more than strength–endurance, and the strength–endurance training further increases strength, power and muscle hypertrophy than the endurance–strength training [85]. The order of exercise in the concurrent training depends on the purpose of the training and the needs of the sport. In addition, the phosphatidylinositol 3-kinase (PI3K)–protein kinase B (AKT)–mammalian target of rapamycin (mTOR) signaling pathways are disrupted when resistance training is performed after glycogen depletion during endurance training [86,87].

Ammar et al. stated in their study that aerobic, anaerobic, and combined training can alter antioxidant status in response to the elevated lipid peroxidation. They stated that under the aerobic and anaerobic conditions, a faster response occurs after training, with higher levels of MDA occurring 5 min after the aerobic training, as well as higher levels of SOD and GPX occurring during anaerobic training (immediately and 5 min after training) and aerobic training (20 min after training). They concluded that the response to oxidative stress depends on the intensity and duration of activity [88]. Mitochondria, in addition to producing ATP during aerobic exercise, appear to be the main intracellular source of pro-oxidants. The mitochondrial electron transfer chain consists of several redox centers, which possibly lead to electron leakage to oxygen and its reduction to O_2_^−^. This is engaged in the dissemination of reactions related to the oxidative chain, which is a progenitor of other ROS [89]. Findings have shown that pro-oxidants of aerobic exercise are much higher than those of anaerobic exercise, and it has been suggested that the response to oxidative stress depends on the type of exercise (such as intensity and duration) [12]. Parker et al. stated that aerobic exercise produces a much higher pro-oxidant status than anaerobic exercise [90,91]. They also stated that increasing the intensity of exercise creates more endogenous antioxidant defenses. These results maybe reflect an enhancement in ROS generation, which stimulates the release of plasma antioxidants and subsequently inhibits ROS with high-intensity exercise [14,64]. High-intensity exercises maybe create redox-related health adaptations by readjusting endogenous antioxidant defenses [62]. A study by Azizbeigi et al. concluded that the endurance, resistance, and concurrent training (endurance + resistance) reduced oxidative stress (MDA) and increased the enzymatic and non-enzymatic antioxidant capacity (SOD, erythrocyte GPx) in untrained men. In addition, TAC levels increased significantly only in the endurance training and the concurrent groups. They stated that it was not clear whether the increase in the enzymatic activity in the concurrent group was due to adaptive effects in response to endurance or resistance training, and it is not clear which one had a greater effect [92].

## 6. Mechanism of the Effect of Creatine Supplementation Combined with Physical Activity on Oxidative Stress

As mentioned, acute and chronic exercises have various effects on oxidative stress. Findings have shown that regular exercise stimulates the endogenous antioxidant system and protects the body against the dangers of oxidative stress. PGC-1α plays a pivotal role in regulating the expression of subunits cytochrome C and cytochrome oxidase in response to a period of treadmill training and long-term training; which indicating that exercise-induced changes in the oxidation capacity are regulated by PGC-1α [88]. Increased expression of PGC-1α is associated with increased expression of nuclear respiratory factor-1 (Nrf-1) and Nrf-2 [70]. In addition to regulating mitochondrial biogenesis, PGC-1α can regulate the expression of endogenous antioxidants in skeletal muscle [90,91]. Nrf-2 is a redox-sensing transcription factor, a major regulator of antioxidants as well as other protective factors responsible for strengthening the antioxidant defense system [82,93]. Additionally, PGC-1α in cell culture can regulate mRNA expression of uncoupling proteins 2 and 3 [94]; this indicates that PGC-1α can increase binding capacity while reducing ROS production in mitochondria [91]. During exercise, several other stimuli are activated that help increase the PGC-1α response; these include increasing cytosolic calcium concentrations, decreasing high-energy phosphate levels and activating AMP-activated protein kinase (AMPK), stimulating the adrenergic system that synthesizes cyclic adenosine monophosphate (c-AMP), and activating protein kinase A and other kinases, including mitogen activated protein kinase (MAPK) [90]. PGC-1α expression appears to be upregulated by ROS. Studies have shown the role of PGC-1α in the increasing of ROS, eliminating enzymes due to elevations in ROS [70]. In skeletal muscle, physical activity upregulates peroxisome proliferator-activated receptors γ (PPARγ)-controlled genes to augment mitochondrial biogenesis, aerobic respiration, and other physical activity-triggered affairs; it has been shown that PPARγ is a coactivator of PGC-1α [71]. A set of adaptations in the body enables PPARγ to regulate antioxidant defense. Evidence confirms that PPARγ is involved in the direct transcriptional regulation of several major endogenous antioxidants [72,73,74]. Unlike chronic physical activity, acute exercise can increase the generation of free radicals and cause oxidative damage to cells. Intensity and duration of physical activity, nutrition, and training status are the main factors influencing oxidative stress caused by physical activity [95]. In addition, aging, dehydration, hypoxia, and gender have many effects on oxidative stress caused by physical activity [96,97,98].

It is clear that enhanced demand for ATP used during exercise enhances ROS levels. Oxidative phosphorylation (OXPHOS) is the main source of ATP production in cells. Changes in the process of increasing ROS production lead to oxidative damage [23]. Endogenous antioxidants could not completely prevent oxidative damage under the physiological and pathological conditions in this case (exercise at altitude). These conditions may disturb the endogenous antioxidant balance and increase oxidative stress. In this case, the use of antioxidant supplements such as creatine can have positive effects on the antioxidant system. Few studies have been performed on the effects of short-term and long-term creatine supplementation along with physical exercise on oxidative stress (Table 1). In this regard, Stefani et al. [99] noted that creatine supplement consumption combined with resistance exercise could reduce oxidative stress (reduced lipoperoxidation in plasma, heart and liver, and gastrocnemius). Moreover, supplementation had positive effects on the SOD activity in all groups. Creatine supplement consumptions possibly have a synergistic effect with resistance training in modulating SOD activity in the heart [99]. In conditions of progressive chronic stress and in resistance training, the supplementation seems to exert a synergistic effect due to the compatibility of resistance training with creatine, which includes the enzymatic compatibility of cellular signaling with SOD in heart tissue. This mechanism happens by the activation of the NADPH oxidase system, which modulates the expression of antioxidant enzymes in a short time through angiotensin II and inflammatory mediators [11,100]. Additionally, the results of Araujo et al. revealed that creatine consumption acts in an additive manner to exercise to raise the antioxidant enzymes in rat livers [101]. Their results showed an increase in glutathione peroxidase (GSH-GPx) activity in the training and training + creatine groups compared to the control group. Regular exercise activates transcription factors (NF-κB and Nrf2), which are responsible for stimulating various genes including mitochondrial GSH-GPx [102,103]. The results of Silva et al. showed that the increase in thiobarbituric acid reactive substance (TBARS) is independent of creatine supplementation [23]. Actually, about 2–5% of the oxygen involved in OXPHOS during physical activity is changed to potentially detrimental oxygen formatives named ROS [104]. Creatine increases intracellular CrP which acts as a cellular energy buffer, thus reducing the OXPHOS dependence on the high-intensity, short-term exercise (Figure 3) [23]. Creatine supplementation may be more effective in short-term training than in long-term training, by reducing intracellular calcium accumulation and limiting ROS formation and reducing oxidative damage [23]. Rahimi stated that consuming 20 g of creatine per day for seven days reduces MDA and 8-hydroxy-2′-deoxyguanosine (8-OHdG) after resistance training. He stated that a resistance exercise using the flat pyramid loading pattern system increases oxidative DNA damage and lipid peroxidation in athletes. Additionally, the antioxidant effects of creatine may be related to its compounds (arginine, glycine and methionine) [40]. Deminice and Jordao concluded that creatine supplement consumption reduces the oxidative stress markers induced by a moderate aerobic exercise [105]. They stated that acute aerobic exercise increases TBARS and total lipid hydroperoxide, and that creatine supplementation can have positive effects on these variables. Mitochondrial protection is very important because this process is required to maintain mitochondrial activity and mitochondriogenesis [106]. As mentioned, creatine has direct antioxidant activity through normalizing mitochondrial mutagenesis, prevents its functional outcomes, and perhaps plays the main role in the stability of mitochondrial activity. Additionally, creatine can prevent mtDNA damage and protect mitochondria by reducing extracellular H_2_O_2_ levels [45,46]. Young et al. reported the capacity of creatine exposure to promote the thiol redox system, of which the GSH and thioredoxin pathways are important components (indirect antioxidant effect) [54]. In addition, studies have shown other indirect antioxidant mechanisms such as hydration and membrane stabilization [5] and increased or normalized cell energy status [107,108]. In contrast, the findings of Kingsley et al. showed that short-term creatine consumption had no effect on the antioxidant defense or protection against lipid peroxidation caused by the exhaustive cycling among healthy men [109]. Deminice et al. stated that creatine supplementation has no effect on the antioxidant parameters; creatine supplement consumption was inadequate to inhibit oxidative stress induced by acute repeated-sprint exercise. They stated that more studies were needed to confirm the antioxidant effects of creatine consumption in humans [110]. Moreover, Percario et al. stated that creatine supplement consumption along with resistance training stimulates oxidative stress and decreases the overall antioxidant capacity [111]. They stated that total antioxidant status (TAS) values in the creatine + training group were significantly decreased compared to the other groups. Considerable enhancement in strength in the creatine + training group may increase the energy production mechanism due to the high capacity for ATP re-synthesis in cells. This condition is maybe suitable for the manifestation of ischemia-reperfusion syndrome, with enhanced uric acid and hydroxyl radical generation causing the mobilization of antioxidant stores (thereby decreasing TAS) to prevent oxidative stress [111].

According to the existing research (Table 1), long-term creatine supplementation along with moderate-intensity resistance and endurance training can probably reduce oxidative stress and increase the antioxidant defense system; however, in the short-term, creatine consumption and its effect on oxidative stress due to endurance exercise is not well known, although it seems that the short-term creatine ingestion possibly reduces oxidative stress due to intense resistance exercise. Considering the antioxidant effects of regular physical activity (PGC-1α, PPARγ) and creatine (maintaining mitochondrial integrity, acting as a cellular energy buffer, reducing extracellular H_2_O_2_ levels, cell membrane stabilization and improvement of cellular energy capacity), it seems that the combined effect of physical activity and creatine consumption can reduce oxidative stress, but further research is needed to conclude more accurately about the intensity of long-term resistance and endurance training with creatine supplementation and the short-term effects of creatine consumption and physical activity on oxidative stress. No research has been done on the effect of creatine supplementation along with the concurrent exercise but considering the antioxidant effects of creatine and the effects of concurrent exercise, it seems that it can have positive effects on oxidative stress. The intensity of exercise, however, can have different effects, and there is a need for more research in this regard [112].

## 7. Conclusions

According to the available information, creatine has antioxidant properties and can be effective through direct and indirect mechanisms. It has a positive effect on oxidative stress and reduces ROS. Creatine can maintain mitochondrial integrity, increase CrP resources, act as a cellular energy buffer, and protect two important cellular targets, mtDNA and RNA, from oxidative damage. In addition, the antioxidant properties of creatine may be related to its constituents (arginine, glycine and methionine) (Figure 3). It seems that creatine consumption combined with long-term training could possibly reduce oxidative stress and improve the antioxidant system. Creatine supplement consumption possibly has a synergistic effect with training, but the intensity and duration of training and supplementation period can play an important role in the antioxidant activity. Not much research has been conducted on the effects of creatine consumption along with long-term and short-term exercise on oxidative stress; therefore, for more accurate conclusions, more research is needed in this field.

## Figures and Tables

**Table 1:** Studies on the effects of short-term and long-term creatine supplementation and exercise on oxidative stress.

Studies	Subject	Exercise	Intervention	Main Outcome
Human study				
Kingsley et al. (2009) [109]	Active males (*n* = 18)	Incremental cycling that was continued until the individualized predetermined point of exhaustion	Ingested 22.8 g·d^−1^ Cr (equivalent to 5 g Cr × 4 daily) for 5 days. Each supplement dose consisted of 5.7 g Cr and 5 g of glucose polymer dissolved in 500 mL of warm water	= Oxidative stress (as measured by serum hydroperoxide concentrations)
Rahimi (2011) [40]	Trained males (*n* = 27)	7 sets, 3–6 repetitions, 80–90% 1RM (bench press, lat pull down, and seated rows)	20 g/day (5 g/serving, 4 serving/day), 7 days before exercise	↓MDA, 8-OHdG
Percario et al. (2012) [111]	Male elite Brazilian handball players (*n* = 26)	5 week RT, 50–95% 1RM, 3–12 repetition	First 5 days: a daily dose of 20 g, remaining 27 days: participants were given a dose of 5 g per day, after training	↓ TAS, =TBARS
Deminice et al. (2013) [110]	Male soccer players (*n* = 25)	2 consecutive running-based anaerobic sprint test, (6 sprints (35 m), maximum speed, 10 s rest between repetition)	0.3 g/kg, 7 days after first exercise	= MDA, GSH, GSH/GSSG ratio, TAC, CAT, SOD, GPX
Animal study				
Deminice and Jordao. (2012) [105]	Male rats (*n* = 64)	1 h swimming with load of 4% of total body weight	2% Cr, 28 days before exercise	↓TBARS, Lipid hydroperoxide ↑GSH/GSSG ratio, TAC = α-Tocopherol, CAT
Silva et al. (2013) [23]	Male rats (*n* = 36)	Exhaustion eccentric running (treadmill, 50–60% VO_2_max, constant velocity 1.0 km/h)	300 mg/kg/day, 15 days, dose of initially: 2 serving/day, dose after 6 days: 1 serving/day	= TBARS, PC, TT, SOD, GPX, CAT
Araujo et al. (2013) [101]	Male Wistar rats (*n* = 40)	25 min treadmill at different fixed speeds for each series, 48 h interval between series, 8 weeks	2% in diet Cr during the maintenance phase equals 20 g·kg^−1^ peak in the phase of 13% were used equivalent to 130 g·kg^−1^	T and TCr groups: ↑H_2_O_2_, GSH-GPx CCr and TCr groups: ↑CAT TCr group: ↓SOD Al groups: GSH, GSH/GSSG
Stefani et al. (2014) [99]	Male Wistar rats (*n* = 40)	8 weeks RT (4 series of 10–12 repetitions, 90 s interval, 4 times per week, 65% to 75% of 1 Concurrent Strength and Aerobic Training Order Influence Training-InduceRM)	The first 7 days prior to the initiation of training: dosage of 0.3 g/kg/day, last 7 weeks: the dosage was set at 0.05 g/kg/day	↓lipoperoxidation, MDA ↑SOD = CAT

= No significant difference; ↓ significantly decreased responses; ↑ significantly increased responses; creatine (Cr); one repetition maximum (1RM); malondialdehyde (MDA); 8-OH-2-deoxyguanosine (8-OH-dG); thiobarbituric acid-reactive substances (TBARS); glutathione (GSH); oxidized glutathione (GSSG); resistance training (RT); total antioxidant capacity (TAC); catalase (CAT); total antioxidant status (TAS); glutathione peroxidase (GSH-GPx); protein carbonyls (PC); total thiol (TT) superoxide dismutase (SOD); glutathione peroxidase (GPX); hydrogen peroxide (H_2_O_2_); training (T); training + creatine (TCr); and control + creatine (CCr).
